# Identification of *ATP8B1* as a Tumor Suppressor Gene for Colorectal Cancer and Its Involvement in Phospholipid Homeostasis

**DOI:** 10.1155/2020/2015648

**Published:** 2020-09-29

**Authors:** Li Deng, Geng-Ming Niu, Jun Ren, Chong-Wei Ke

**Affiliations:** ^1^Department of General Surgery, The Fifth People's Hospital of Shanghai, Fudan University, Shanghai, China; ^2^Department of General Surgery, The Shanghai Public Health Clinical Center, Fudan University, Shanghai, China

## Abstract

Homeostasis of membrane phospholipids plays an important role in cell oncogenesis and cancer progression. The flippase ATPase class I type 8b member 1 (ATP8B1), one of the P4-ATPases, translocates specific phospholipids from the exoplasmic to the cytoplasmic leaflet of membranes. *ATP8B1* is critical for maintaining the epithelium membrane stability and polarity. However, the prognostic values of *ATP8B1* in colorectal cancer (CRC) patients remain unclear. We analyzed transcriptomics, genomics, and clinical data of CRC samples from The Cancer Genome Atlas (TCGA). *ATP8B1* was the only potential biomarker of phospholipid transporters in CRC. Its prognostic value was also validated with the data from the Gene Expression Omnibus (GEO). Compared to the normal group, the expression of *ATP8B1* was downregulated in the tumor group and the CRC cell lines, which declined with disease progression. The lower expression level of *ATP8B1* was also significantly associated with worse survival outcomes in both the discovery samples (359 patients) and the validation samples (566 patients). In multivariate analyses, low *ATP8B1* levels predicted unfavorable OS (adjusted HR 1.512, 95% CI: 1.069-2.137; *P* = 0.019) and were associated with poor progress-free interval (PFI) (adjusted HR: 1.62, 95% CI: 1.207-2.174; *P* = 0.001). The pathway analysis results showed that the underexpression of *ATP8B1* was negatively associated with phospholipid transport, phospholipid metabolic process, and cell-cell adherent junction and positively associated with the epithelial-mesenchymal transition in CRC. Our analysis suggests that *ATP8B1* is a potential cancer suppressor in CRC patients and may offer new strategies for CRC therapy.

## 1. Introduction

Colorectal cancer (CRC) is the third most common cancer and the fourth leading cause of cancer-related deaths globally [1]. Early detection and improved treatment strategies decrease the total CRC mortality [2]. However, the five-year relative survival rate of patients with metastatic disease is estimated at between 10% and 14% [2]. Alteration of the somatic copy number, CpG island hypermethylation, and microsatellite instability are the major relative mechanisms associated with poor prognosis in CRC patients [3]. Therefore, biomarkers are vital for the diagnosis, prognostication, and development of anticancer drugs for CRC patients [4].

Membrane phospholipid homeostasis plays a vital role in several biological processes. This depends on three transporter families, including P4-ATPases (flippases), ATP-binding cassette (ABC) transporters (floppases), and scramblases [5]. Decreased expression of flippases causes abnormal membrane phospholipid distribution, thereby enhancing tumor progression and metastasis [6]. The subsets of ABC transporters (ABCB1, ABCC1, and ABCG2) have widely been reported as multidrug efflux pumps conferring tumor resistance to chemotherapy [7]. The deficiency of either *ABCB4* or *ATP11C* (a member of P4-ATPases) is associated with liver oncogenesis [8, 9]. Upregulated *PLSCR1* expression (a member of scramblases) can inhibit tumor growth and proliferation [10]. The genetic alteration of *ATP8B1* has also been detected in CRC and hepatocellular carcinoma [11, 12]. However, the prognostic role of membrane phospholipid transporters in CRC has not been elucidated. This study explored the expression of all the 31 genes coding for phospholipid transporters and evaluated the associations with prognosis in CRC using the data from The Cancer Genome Atlas (TCGA) and Gene Expression Omnibus (GEO) datasets. Our analysis indicates that *ATP8B1* may be a novel oncogene suppressor in CRC patients. The correlation of *ATP8B1* with the normal function of membrane phospholipids and proteins coupled with its underexpression could be a valuable indicator of unfavorable clinical outcomes of CRC patients.

## 2. Methods

### 2.1. Data Acquisition

The TCGA pan-cancer data for Toil RNA sequencing Recompute was downloaded from the UCSC Xena browser [13] (https://xenabrowser.net/datapages/). Gene expression was normalized to eliminate computational batch effects [13]. All gene expressions were transformed using a log2 TPM scale. Approximately 382 colorectal tumors and 51 normal colorectal samples were obtained from the TCGA pan-cancer data to identify the DEGs for phospholipid transporters, including 32 matched tumors and normal samples. High-quality survival data was also downloaded from the UCSC Xena browser [14]. After excluding data with incomplete follow-up information, 359 of 382 CRC patients were selected for further analysis. The microarray dataset (GSE39582) with clinical features was obtained from the GEO (https://www.ncbi.nlm.nih.gov/geo/) to validate the retrieved information. GSE39582 was measured using the Affymetrix Human Genome U133 Plus 2.0 Array. All the raw data for mRNA expression were normalized using the Limma R package and transformed into a log2 scale. The probe showing the highest expression level was selected for scenarios where two or more probes were annotated to one gene symbol. The tidy RNASeq and protein spectra data were obtained from Wang et al. [15]. They were used to identify different *ATP8B1* expressions in 44 CRC cell lines by comparing the TCGA colorectal tumor and normal tissue samples. Informative data on gene mutations for KRAS, BRAF, and TP53 and microsatellite instability in TCGA were obtained from the Genomic Data Commons (GDC) through the TCGAbiolinks R package [16]. The GSE39582 data also contained this information. Subtypes of Consensus Molecular Subtypes (CMS) were obtained by calculating transcriptome data from the CMScaller R package [17].

### 2.2. Identifying the Differentially Expressed Genes for Phospholipid Transporters

All the transporter genes for P4-ATPases, ABC transporters, and scramblases were obtained from previous reports [5, 18]. The differentially expressed genes (DEGs) were identified using the Wilcoxon signed-rank test and false discovery rate (FDR). The cutoff values were determined based on the FDR < 0.05 and ∣log2 fold change (FC) | >1.

### 2.3. Online Database

The Oncomine database (https://www.oncomine.org/resource/login.html) was used to explore the *ATP8B1*expression level in previous studies [19]. The following parameters were used: “cancer vs. normal analysis,” “gene summary view,” *P* value of 0.01, fold change of 1.5, and top 10% gene rank. The relationship between *ATP8B1* expression and genomic details in CRC was displayed through the cBioportal (http://www.cbioportal.org/) [20]. Besides, the differences in *ATP8B1* DNA methylation on MEXPRESS (https://mexpress.be/index.html) were determined between colorectal tumors and normal tissues [21]. The results for cBioportal and MEXPRESS were calculated from the TCGA CRC data.

### 2.4. Gene Set Enrichment Analysis and Gene Ontology Analysis of *ATP8B1*-Related Genes in CRC

Gene set enrichment analysis (GSEA) of TCGA colorectal data was performed using the clusterProfiler R package [22]. The Kyoto Encyclopedia of Genes and Genomes (KEGG) and the Broad Molecular Signatures Database (MSigDB v7.1) hallmark gene sets (set H, 50 gene sets) were used [23]. Furthermore, *ATP8B1* significantly related genes (217 genes) in TCGA and GSE39582 dataset were selected for Gene Ontology (GO) analysis using the clusterProfiler R package. The default-weighted statistic was considered significantly enriched at 1000 times permutations with *P* < 0.05. The results were generated using the clusterProfiler and ComplexHeatmap R package.

### 2.5. Statistical Analysis

All statistical analyses were performed using the R software 3.6.2. A *P* value < 0.05 was considered statistically significant. A two-tailed Wilcoxon test was used to determine significant differences between the subgroups. To analyze the survival differences, patients were categorized into a “high” or “low” group based on the *ATP8B1* best-cutoff expression using the MaxStat R package (maximally selected rank statistics) [24]. The Kaplan-Meier survival curves were generated between the high-expression group and the low-expression group. The curves were compared using a log-rank test. Stratified survival analysis results were discussed based on clinicopathological features. Univariate and multivariate Cox proportional hazards regression models were designed to estimate whether *ATP8B1* expression was the independent prognostic value in TCGA or GSE39582 datasets.

## 3. Results

### 3.1. Patient Demographics

A total of 925 patients were engaged in the prognostic study. The clinicopathological features, including gender, age, American Joint Committee on Cancer (AJCC) 7th edition staging, and mutational status of KRAS/BRAF/TP53, of discovery and validation sets, are shown in Table 1. The TCGA cohort of 359 patients was the discovery set while the GSE39582 cohort of 566 patients was the validation set. In the two cohorts, the median age of patients was 65 years. The ratio of each stage of patients was similar. However, the median OS (overall survival) and RPS (relapse-free survival) in the validation set (51 months and 43 months) were longer than those of the discovery set (22.4 months and 19.6 months).

### 3.2. Association between Phospholipid Transporter DEGs and Survival Outcomes

**Figure 1:**
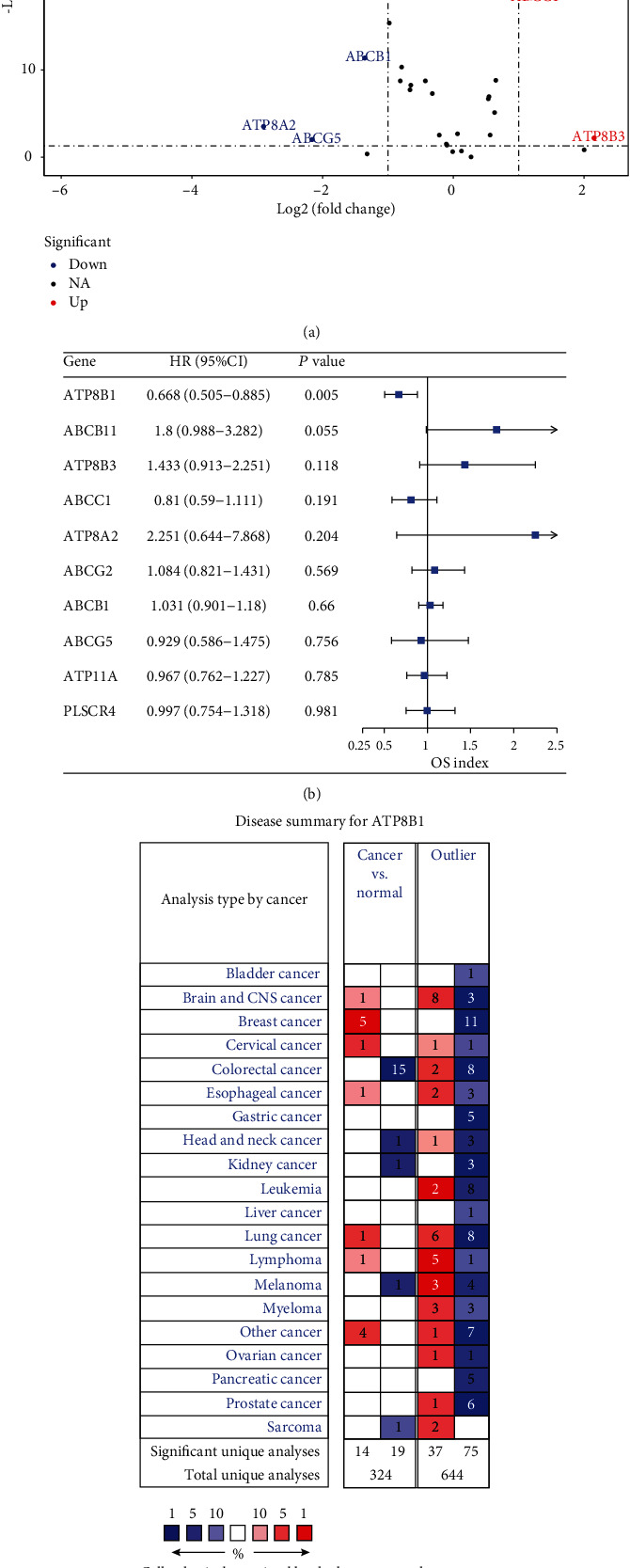
(a) Differential expression of 31 genes for P4-ATPases, ABC transporters, and scramblases in CRC tissue samples (*n* = 382) compared with normal colorectal samples (*n* = 51) is shown in the –log (FDR) vs. log (FC) plot. The red dots represent 3 upregulated genes, the blue dots represent 7 downregulated genes, and the remaining black dots represent genes that are not differentially expressed in CRC samples. (b) Hazard ratios and 95% confidence interval of the differentially expressed genes for the overall survival (filtered by *P* < 0.05). (c) *ATP8B1* expression showing differences between cancer and normal tissues in various tumors using Oncomine. The cell color is determined by the best gene rank percentile for analysis within the cell.

A total of 31 genes expressed in phospholipid transporters were analyzed from 382 CRC and 51 normal colorectal tissue samples (Table S1). The patient characteristics are shown in Table S2. Using the Wilcoxon test, 10 differentially expressed genes were identified (Figure 1(a)). Furthermore, *ATP8B1* genes were selected using univariate Cox regression duo with a significant *P* value of <0.01 (Figure 1(b)). The *ATP8B1* expression had been reported in hepatocytes [25] and different epithelial cell types [26]. The results from the Oncomine database search revealed that *ATP8B1* expression was downregulated in different CRC cohorts (Figure 1(c)). In the TCGA-matched tumor and normal samples, 9 DEGs were identified (Figure S1A). The *ATP8B1* expression was also lower in the tumor group (Figure S1B). Besides, the mRNA and protein mean expressions of *ATP8B1* were lower in 44 CRC cell lines and 90 TCGA CRC samples compared with the 60 colorectal normal samples (Figures S1C and S1D).

### 3.3. Prognostic Value of *ATP8B1* in the TCGA Set

**Figure 2:**
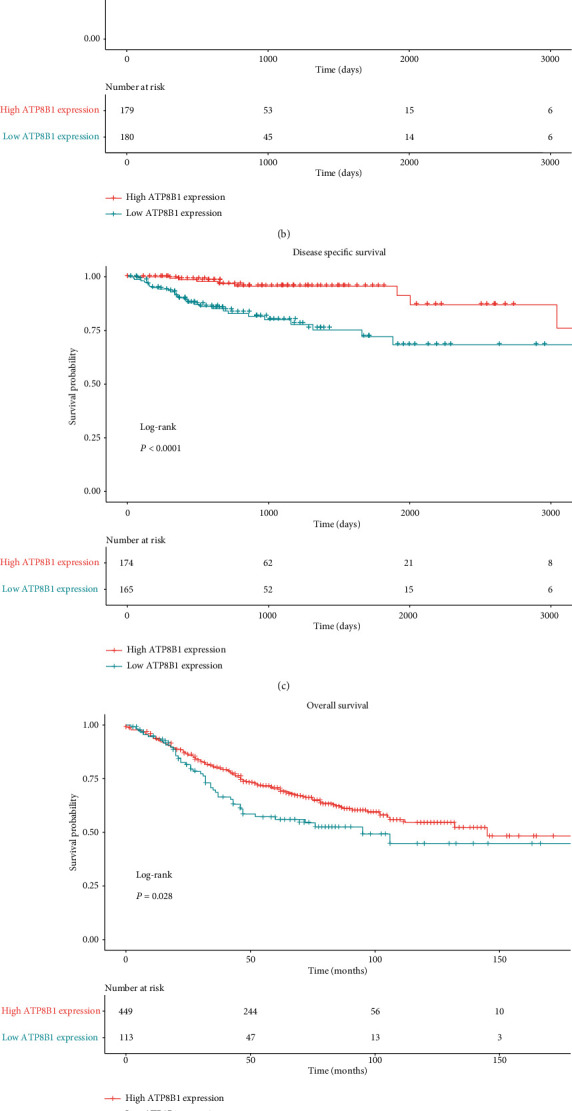
(a, c) Correlation between the overall survival, progress-free interval, and disease-specific survival and *ATP8B1* expression in the TGCA CRC dataset. *ATP8B1* expression was transformed by log2 (TPM + 0.001). (d, e) Correlation between overall survival and relapse-free survival and *ATP8B1* expression in the validation dataset. *ATP8B1* expression was transformed by a log2 scale.

**Figure 3:**
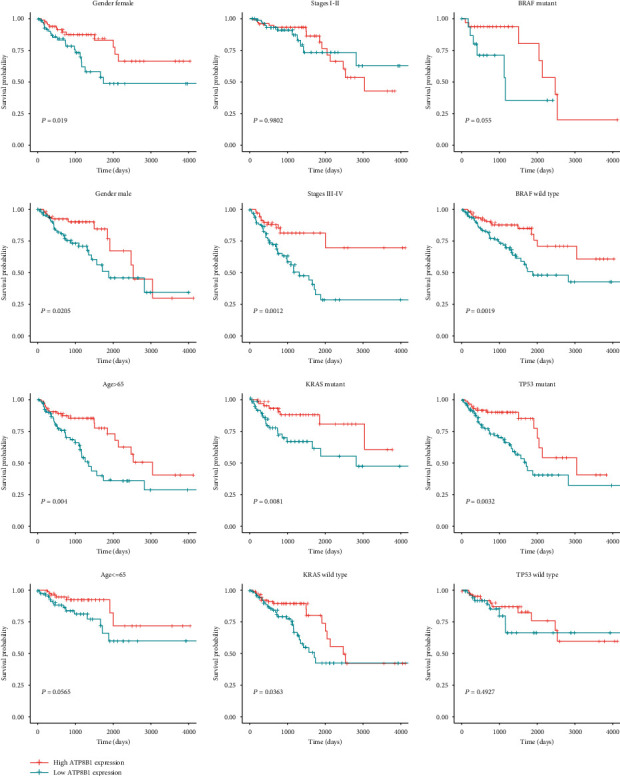
Subgroup analysis of OS based on gender, age, stage, KRAS mutation status, BRAF mutation status, and TP53 mutation status in the TCGA CRC dataset.

**Figure 4:**
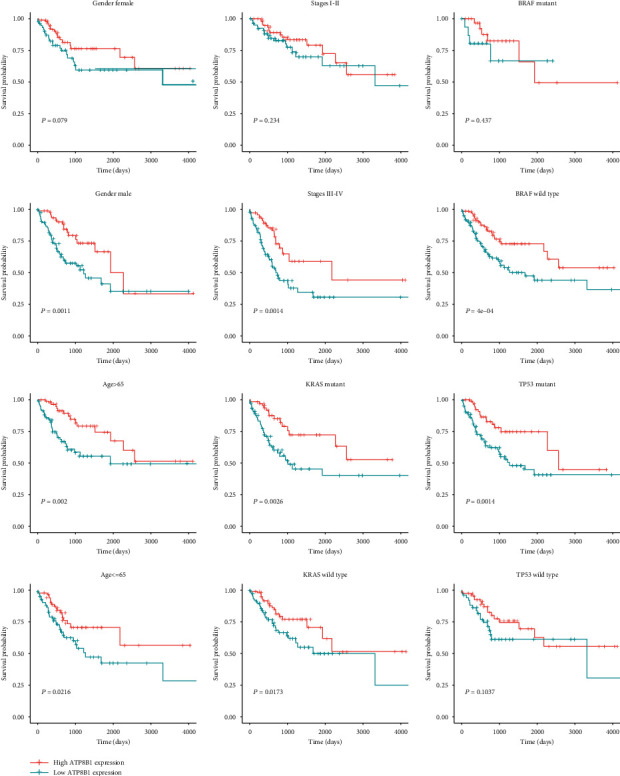
Subgroup analysis of PFI based on gender, age, stage, KRAS mutation status, BRAF mutation status, and TP53 mutation status in the TCGA CRC dataset.

Based on the MaxStat best-cutoff method, patients were divided into high or low *ATP8B1* expression groups. The low-expression group had a poor OS (*P* < 0.001), PFI (*P* < 0.001), and DSS (disease-specific survival) (*P* < 0.001) compared with the high-expression group (Figures 2(a)–2(c)). Multivariate Cox regression analysis was used to explore the relationship between *ATP8B1* expression and survival outcome in different clinicopathological features. *ATP8B1* was considered the independent favorable prognostic value after adjustment of significant clinical factors (OS: age of 65, stages III-IV vs. stages I-II, TP53 mutant status; PFI: stages III-IV vs. I-II) in the univariate Cox analysis (Table 2). Low *ATP8B1* level was associated with shorter OS with an adjusted hazard ratio (HR) of 1.512 (95% CI: 1.069-2.137; *P* = 0.019) and a worse PFI (adjusted HR: 1.62, 95% CI: 1.207-2.174; *P* = 0.001). Assessment of the OS and PFI subgroups of CRC patients was also performed. In CRC subgroup evaluation, the Kaplan-Meier curve results showed that gender (male/female), age (older, >65), stages III-IV, KRAS mutation, KRAS wild type, BRAF wild type, and TP53 mutation in the low *ATP8B1* expression group were closely associated with poor OS (Figure 3). In addition, gender (male), age (younger, ≤65 years; older, >65 years), stages III-IV, KRAS mutation, KRAS wild type, BRAF wild type, and TP53 mutation in the low *ATP8B1* expression group were associated with poor PFI (Figure 4).

### 3.4. Prognostic Analysis of the Validation Set

The GSE39582 patients were also categorized into high or low *ATP8B1* expression groups based on the cutoff point. The low *ATP8B1* expression group was significantly correlated with worse OS (*P* = 0.028) and RFS (*P* = 0.003) as shown in Figures 2(d) and 2(e). In the multivariate Cox regression model, low *ATP8B1* expression was correlated with shorter RFS (adjusted HR: 1.464, 95% CI: 1.089-1.969; *P* = 0.012). This was after adjustment for stages III-IV vs. I-II and KRAS mutation status; however, it was not independent in OS (Table 3). The CRC subgroup assessment showed that gender (female), age (older, >65), and TP53 wild type in the low *ATP8B1* expression group were associated with poor OS (Figure S2). Moreover, gender (female/male), age (older, >65), KRAS wild type, and BRAF wild type in the low *ATP8B1* expression group were associated with poor RFS (Figure S3).

### 3.5. Genetic Alteration of *ATP8B1* in CRC Tissues

To explore the association between *ATP8B1* downregulation and genetic alteration, the mutation status and methylation levels were, respectively, obtained from the cBioportal and MEXPRESS. The results showed barely 6% *ATP8B1* mutation frequency in the TCGA CRC, which was independent of its expression level (Figure S4A). Then, we stratified the CRC patients into four groups based on the *ATP8B1* copy number values using GISTIC2. As shown in Figure S4B, the CRC samples harboring *ATP8B1* deletion exhibited lower mRNA expression than those that exhibited diploid genes. Besides, MEXPRESS provided a visual interface for the methylation level of the *ATP8B1* promoter region (Figure S4C). It was revealed that the CpG islands were hypomethylated. However, the low copy number variation of chromosome 18 was associated with the downregulated *ATP8B1* expression level (Figure S4A).

### 3.6. The Biological Pathways Associated with *ATP8B1* in TCGA CRC Profiles

**Figure 5:**
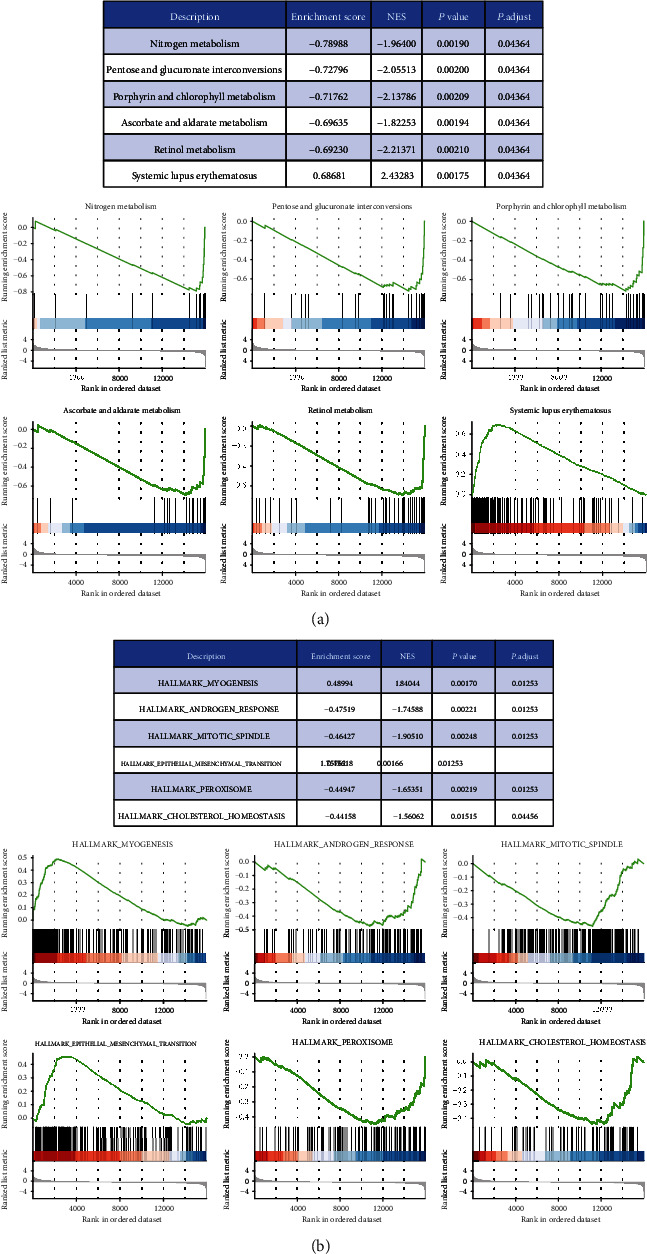
GSEA of differential genes between the low- and high-expression groups of *ATP8B1* was performed, and the top six items of significant enrichment scores were identified. The left side represents the *ATP8B1* low-expression group, and the right side represents the *ATP8B1* high-expression group in each GSEA diagram: (a) KEGG pathway gene sets and (b) hallmark gene sets.

**Figure 6:**
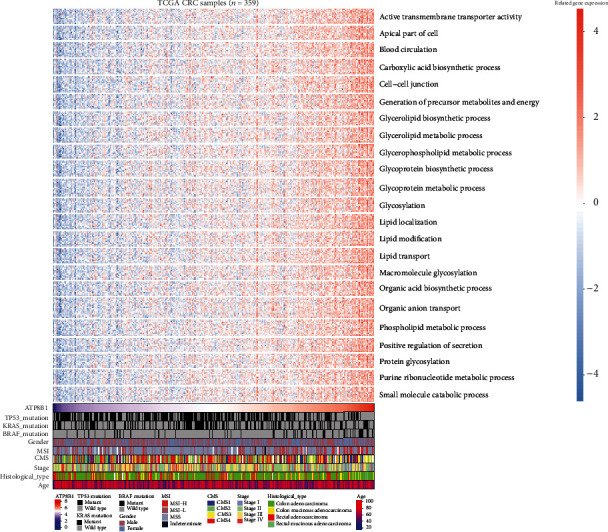
A heatmap of the involved pathways for *ATP8B1*-related genes and clinicopathological features in TCGA CRC samples.

**Figure 7:**
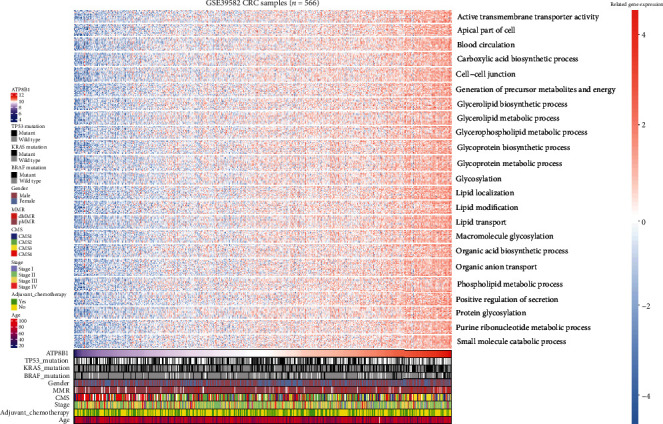
A heatmap of the involved pathways for *ATP8B1*-related genes and clinicopathological features in the GSE39582 dataset.

To explore the pathway changes in CRC influenced by *ATPB1* downregulation, we performed GSEA using the KEGG pathway gene sets and MSigDB hallmark gene sets. The results revealed that a large number of gene sets were enriched in high-expression *ATP8B1* samples compared with low-expression *ATP8B1* samples. Among the six most significantly enriched KEGG pathway gene sets, five pathways related to lipid metabolism activities (nitrogen metabolism, pentose and glucuronate interconversions, porphyrin and chlorophyll metabolism, ascorbate and aldarate metabolism, and retinol metabolism) were highly enriched in high-expression *ATP8B1* patients (Figure 5(a)). Similarly, among the six most significantly enriched hallmark gene sets, genes involved in cholesterol homeostasis were highly enriched in high-expression *ATP8B1* patients (Figure 5(b)). We also found genes involved in myogenesis and epithelial-mesenchymal transition (EMT) which were highly enriched in low-expression *ATP8B1* patients (Figure 5(b)). Furthermore, particular genes always had similar expression changes in the same physiological process. Pearson's correlation analysis was performed to select genes that were strongly correlated with *ATP8B1* (Pearson's *R* ≥ 0.4 in TCGA and GSE39582), to explore the potential biological functions affected by *ATP8B1* expression. Based on the cutoff criteria, 1023 and 485 genes were selected from the TCGA and GSE39582 datasets, respectively. The overlapped genes (217 genes) in the two datasets were selected for GO analysis. The results revealed that *ATP8B1* expression was enriched in phospholipid transport, phospholipid metabolic process, and cell-cell adherent junction pathways (Figures 6 and 7). It was also observed that patients with advanced stages (stages III-IV; Figure 6) and CMS4 (Figure 7) mainly exhibited low *ATP8B1* expression (advanced stages: chi‐square = 6.1937, *P* value = 0.01282; CMS4: chi‐square = 22.663, *P* value < 0.0001). From the TCGA data, the BRAF mutant was more common in higher *ATP8B1* expression cases (BRAF mutant: chi‐square = 6.3888, *P* value = 0.01148), whereas the other molecular status (KRAS or TP53) did not show any difference. This highlighted that lower *ATP8B1* expression may be associated with malignancy and poor survival rates in CRC patients.

## 4. Discussion

Colorectal cancer is a heterogeneous disease classified into four molecular subtypes based on the large-scale gene expression analysis [3]. This classification, however, can hardly be leveraged for clinical use. Patients with identified TNM stages are characterized by different prognostic outcomes [27]. Therefore, the identification of biomarkers to stratify potential high-risk patients with different prognoses is crucial. This study examined *ATP8B1* expression in CRC and also reported its downregulation as an independent prognostic biomarker for unfavorable survival outcomes of CRC patients. In CRC samples and cell lines, both the RNA and protein expressions of *ATP8B1* declined (Figures S1C and S1D). The loss of *ATP8B1* expression was correlated with stage progression (Figure 6).

In this study, lower *ATP8B1* expression was a prognostic factor for predicting worse CRC outcomes in stratified survival analyses of the advanced stage, older age, and other molecular features. In addition, TP53, KRAS, and BRAF influenced CRC patient outcomes [28]. It is necessary to emphasize that stratified survival analyses of *ATP8B1* have no prognostic value in the early stage or TP53 wild-type subgroup. This could be due to the higher expression of *ATP8B1* in the early stages and TP53 wild type, which was considered a protective factor for prognosis (Table 2). However, further research exploring the underlying mechanism is essential. Differences in stratified survival analyses between the two datasets may be due to the specificity, randomness, or noise of each data. KRAS wild type was determined to be associated with favorable OS/RFS using the validation data (Table 3). However, the correlation between the mutational status of BRAF or KRAS and CRC prognosis remains unclear [29]. This could be due to variation in patients, statistical methods, and available covariates.

*ATP8B1* was abundantly expressed in the gastrointestinal tract, and its downregulated expression led to aberrant microvilli morphology [26]. This has been studied in benign recurrent intrahepatic cholestasis type 1 (BRIC1) and progressive familial intrahepatic cholestasis type 1 (PFIC1) [30]. Degradation at either RNA or protein level could be caused by gene mutations. However, our study reported *ATP8B1* mutation frequency at 6%, which was not correlated with the expression level in CRC patients (Figure S4A). Besides, RNA downregulation could be ascribed to the hypermethylation of tumor suppressor genes [31]. We, therefore, investigated the methylation status of CpG sites within the *ATP8B1* promoter sequence. Using the TCGA data, *ATP8B1* promoter hypermethylation was not detected in the CRC samples (Figure S4C). Therefore, *ATP8B1* mutations and promoter hypermethylation inadequately explain the low *ATP8B1* expression in CRC patients. Interestingly, chromosome 18q of most CRC patients has lost the diploid copy number where the *ATP8B1* is located (Figure S4A). A correlation between ATP8B1 copy number deletion and mRNA underexpression was also found among the CRC samples (Figure S4B). The major genetic variance in CRC is caused by chromosomal instability due to aneuploidy and loss of heterozygosity [32]. Moreover, previous studies have reported various CRC forms caused by the loss of heterozygosity on chromosome 18q [11, 33]. We speculated that decreased *ATP8B1* expression might be due to genomic deletion; however, an in-depth analysis is needed to fully explore this mechanism.

Membrane phosphatidylserine is translocated by ATP8B1 from the outer layer to the inner layer of the cell membrane [34]. This plays an important role in cell membrane stabilization, phospholipid metabolism and transport, and signal transduction. Overexpression of phosphatidylserine in the outer cell membrane layer has previously been reported in many cancers [35]. This is due to the low activity of flippases such as *ATP8B1* and is related to poor outcomes [6, 35]. Our present evidence revealed that a correlation between the downregulation of *ATP8B1* expression and low activity of phospholipid-related metabolism or transport pathways was essential for maintaining membrane phospholipid function (Figures 5–7). Besides, apical cytoskeleton disorganization and defect in apical membrane proteins were found in *ATP8B1*-deficient cells [36]. During cancer progression, cells lose epithelial architecture and polarity due to decreased levels of membrane proteins [37]. Moreover, the loss of cell polarity increased the EMT activity, which was associated with metastasis and poor prognosis in CRC [38]. In our GSEA results, we also observed that the EMT pathway was enriched in the lower *ATP8B1* expression group (Figure 5). Similarly, the function of the cell-cell junction was positively associated with the *ATP8B1* expression level in CRC (Figures 6 and 7). Conversely, the CMS4 type was associated with CRC unfavorable outcomes [3]. These were potentially related to the lower *ATP8B1* expression (Figure 7). This study, therefore, hypothesized that *ATP8B1* expression deficiency might lead to the abnormal distribution or metabolism of the membrane phospholipids and the malfunction of membrane junction proteins, thereby enhancing CRC progression. This study was limited by the lack of an antibody to verify protein expression of *ATP8B1* in the CRC samples. Besides, mRNA expression is not a locally available clinical parameter due to the high cost of storage and fresh tissue processing. Other methods for detecting *ATP8B1* protein expression in CRC should be explored. Moreover, superior results could be achieved with stable mRNA expression obtained from formalin-fixed, paraffin-embedded tissue samples at a low cost.

## 5. Conclusion

In conclusion, the downregulation of *ATP8B1* is associated with advanced stages, adverse molecular types, and poor outcomes in CRC patients. Moreover, *ATP8B1* expression positively regulates phospholipid localization, metabolism, and intercellular adhesion function. These findings improve our understanding of malignant progression in CRC patients. Although the precise molecular mechanisms remain indefinite, *ATP8B1* gene expression has shown great potential in predicting the prognosis and treatment of CRC patients.

## Figures and Tables

**Table 1:** Patient demographics.

	Discovery dataset (TCGA CRC, *n* = 359)	Validation dataset (GSE39582, *n* = 566)
Gender		
Male	194 (54%)	310 (55%)
Female	165 (46%)	256 (45%)
Age (years)		
Median (IQR)	66 (55-74.5)	68.1 (59-76)
>65 vs. ≤65	184 vs. 175	342 vs. 124
Stage		
I	56	37^∗^
II	134	264
III	116	205
IV	53	60
KRAS status		
Wild type	217 (60%)	328 (58%)
Mutant	142 (40%)	217 (39%)
N/A	—	21 (3%)
BRAF status		
Wild type	307 (86%)	461 (82%)
Mutant	52 (14%)	51 (9%)
N/A	—	54 (9%)
TP53 status		
Wild type	140 (39%)	161 (28%)
Mutant	219 (61%)	190 (34%)
N/A	—	215 (38%)
OS		
Median	22.4 (months)	51 (months)
Range	0-150 (months)	0-201 (months)
Event/nonevent	77/282	191/371
PFI or RFS^∗∗^		
Median	19.6 (months)	43 (months)
Range	0-150 (months)	0-201 (months)
Event/nonevent	100/259	177/380

^∗^Stage I group included four stage 0 cases in the GSE38582 dataset. ^∗∗^PFI (progress-free interval) was provided in the TCGA CRC cohort, while RFS (relapse-free survival) was obtained in GSE38582.

**Table 2:** Univariate and multivariate analyses of factors associated with overall survival and progress-free interval in the TCGA CRC cohort.

	Univariate analysis^b^		Multivariate analysis^b^	
	HR (95% CI)	*P* value^a^	HR (95% CI)	*P* value^a^
TCGA CRC/OS (*n* = 359)				
Gender (male vs. female)	1.143 (0.83-1.574)	0.412		
Age (≤65 vs. >65)	**0.586 (0.415-0.827)**	**0.002**	**0.522 (0.369-0.739)**	**<0.001**
Stage (III-IV vs. I-II)	**2.077 (1.484-2.908)**	**<0.001**	**2.058 (1.457-2.906)**	**<0.001**
KRAS (wild type vs. mutant)	1.125 (0.812-1.558)	0.479		
BRAF (wild type vs. mutant)	0.842 (0.552-1.285)	0.425		
TP53 (wild type vs. mutant)	**0.65 (0.456-0.927)**	**0.017**	0.774 (0.536-1.119)	0.173
ATP8B1 expression (low vs. high)	**1.743 (1.247-2.435)**	**0.001**	**1.512 (1.069-2.137)**	**0.019**
TCGA CRC/PFI (*n* = 359)				
Gender (male vs. female)	1.292 (0.97-1.722)	0.08		
Age (≤65 vs. >65)	1.168 (0.884-1.542)	0.275		
Stage (III-IV vs. I-II)	**2.132 (1.587-2.864)**	**<0.001**	**2.029 (1.509-2.729)**	**<0.001**
KRAS (wild type vs. mutant)	0.873 (0.66-1.154)	0.339		
BRAF (wild type vs. mutant)	1.315 (0.828-2.087)	0.246		
TP53 (wild type vs. mutant)	0.798 (0.595-1.07)	0.131		
ATP8B1 expression (low vs. high)	**1.739 (1.298-2.331)**	**<0.001**	**1.62 (1.207-2.174)**	**0.001**

^a^Cohorts were calculated using the Cox proportional hazards regression model. Bold indicates statistically significant values. ^b^Multivariate analysis used the covariates associated with survival in univariate models (*P* < 0.05). The Wald statistic was applied to test whether the covariates were independent variables (*P* < 0.05). Bold indicates statistically significant values.

**Table 3:** Univariate and multivariate analyses of factors associated with overall survival and relapse-free survival in the GSE39582 cohort.

	Univariate analysis^b^		Multivariate analysis^b^	
	HR (95% CI)	*P* value^a^	HR (95% CI)	*P* value^a^
GSE39582/OS (*n* = 566)				
Gender (male vs. female)	1.21 (0.986-1.486)	0.068		
Age (≤65 vs. >65)	**0.763 (0.616-0.945)**	**0.013**	**0.748 (0.602-0.93)**	**0.009**
Stage (III-IV vs. I-II)	**1.496 (1.221-1.832)**	**<0.001**	**1.483 (1.205-1.825)**	**<0.001**
KRAS (wild type vs. mutant)	**0.804 (0.655-0.987)**	**0.037**	0.823 (0.67-1.011)	0.063
BRAF (wild type vs. mutant)	0.928 (0.645-1.336)	0.688		
TP53 (wild type vs. mutant)	0.881 (0.688-1.127)	0.313		
ATP8B1 expression (low vs. high)	**1.297 (1.028-1.638)**	**0.028**	1.207 (0.953-1.53)	0.119
GSE39582/RFS (*n* = 566)				
Gender (male vs. female)	1.182 (0.956-1.463)	0.123		
Age (≤65 vs. >65)	1.117 (0.905-1.379)	0.301		
Stage (III-IV vs. I-II)	**2.067 (1.658-2.577)**	**<0.001**	**1.934 (1.546-2.421)**	**<0.001**
KRAS (wild type vs. mutant)	**0.781 (0.63-0.967)**	**0.023**	0.814 (0.657-1.008)	0.06
BRAF (wild type vs. mutant)	1.051 (0.703-1.572)	0.809		
TP53 (wild type vs. mutant)	0.804 (0.627-1.031)	0.085		
ATP8B1 expression (low vs. high)	**1.541 (1.153-2.059)**	**0.003**	**1.464 (1.089-1.969)**	**0.012**

^a^Cohorts were calculated using the Cox proportional hazards regression model. Bold indicates statistically significant values. ^b^Multivariate analysis used the covariates associated with survival in the univariate models (*P* < 0.05). The Wald statistic was applied to test whether the covariates were independent variables (*P* < 0.05). Bold indicates statistically significant values.

## Data Availability

The datasets of the current study are downloaded from TCGA and GEO, which are available in Tables S3–S6.

## Supplementary Materials

Supplementary Materials Table S1: gene lists of P4-ATPases, ABC transporters, and scramblases. Table S2: patient demographics of DEG analysis. Table S3: TCGA RNASeq data for 382 colorectal tumor and 51 normal colorectal samples. Table S4: clinical and pathological data of TCGA 359 colorectal tumor samples. Table S5: GSE39582 microarray data for 566 colorectal tumor samples. Table S6: clinical and pathological data of GSE39582. Figure S1: (A) the differential expression of 31 genes for P4-ATPases, ABC transporters, and scramblases in matched TCGA colorectal tumor and normal samples (*n* = 32) is shown in the –log (FDR) vs. log (FC) plot. The red dots represent 4 upregulated genes, the blue dots represent 5 downregulated genes, and the remaining black dots represent genes that are not differentially expressed. (B) The different RNA ATP8B1 expression between paired CRC tumor and normal samples. (C) The different RNA ATP8B1 expression between 44 CRC cell lines, 95 tumors, and 60 normal tissues. (D) The different protein spectral ATP8B1 counts between 44 CRC cell lines, 95 tumors, and 60 normal tissues. (∗ means *P* < 0.05 and ∗∗∗∗ means *P* < 0.0001). Figure S2: subgroup analysis of OS based on gender, age, stage, KRAS mutation status, BRAF mutation status, and TP53 mutation status in GSE39582 data. Figure S3: subgroup analysis of RFS based on gender, age, stage, KRAS mutation status, BRAF mutation status, and TP53 mutation status in GSE39582 data. Figure S4: (A) a heatmap between ATP8B1 expression and genetic alteration including copy number status of chromosome 18q from the cBioportal (526 TCGA colorectal cancer samples). (B) The relationship between ATP8B1 copy number variations and gene expressions of 382 TCGA colorectal samples from the UCSC Xena browser (high-level deletion: -2, low-level deletion: -1, diploid: 0, low-level amplification: 1). (C) The methylation level of the ATP8B1 promoter region in TCGA colorectal tumor and normal samples from the MEXPRESS. (COAD: 461 primary tumor samples and 93 normal samples; READ: 172 primary tumor samples and 19 normal samples).
