# Supplementary material for: Identification of ATP8B1 as a Tumor Suppressor Gene for Colorectal Cancer and Its Involvement in Phospholipid Homeostasis
Source: Biomed Res Int. 2020 Sep 29;2020:2015648. doi: 10.1155/2020/2015648 (PMC7542516; doi:10.1155/2020/2015648)
Supplement: Supplementary Materials — Table S1: gene lists of P4-ATPases, ABC transporters, and scramblases. Table S2: patient demographics of DEG analysis. Table S3: TCGA RNASeq data for 382 colorectal tumor and 51 normal colorectal samples. Table S4: clinical and pathological data of TCGA 359 colorectal tumor samples. Table S5: GSE39582 microarray data for 566 colorectal tumor samples. Table S6: clinical and pathological data of GSE39582. Figure S1: (A) the differential expression of 31 genes for P4-ATPases, ABC transporters, and scramblases in matched TCGA colorectal tumor and normal samples (n = 32) is shown in the –log (FDR) vs. log (FC) plot. The red dots represent 4 upregulated genes, the blue dots represent 5 downregulated genes, and the remaining black dots represent genes that are not differentially expressed. (B) The different RNA ATP8B1 expression between paired CRC tumor and normal samples. (C) The different RNA ATP8B1 expression between 44 CRC cell lines, 95 tumors, and 60 normal tissues. (D) The different protein spectral ATP8B1 counts between 44 CRC cell lines, 95 tumors, and 60 normal tissues. (∗ means P < 0.05 and ∗∗∗∗ means P < 0.0001). Figure S2: subgroup analysis of OS based on gender, age, stage, KRAS mutation status, BRAF mutation status, and TP53 mutation status in GSE39582 data. Figure S3: subgroup analysis of RFS based on gender, age, stage, KRAS mutation status, BRAF mutation status, and TP53 mutation status in GSE39582 data. Figure S4: (A) a heatmap between ATP8B1 expression and genetic alteration including copy number status of chromosome 18q from the cBioportal (526 TCGA colorectal cancer samples). (B) The relationship between ATP8B1 copy number variations and gene expressions of 382 TCGA colorectal samples from the UCSC Xena browser (high-level deletion: -2, low-level deletion: -1, diploid: 0, low-level amplification: 1). (C) The methylation level of the ATP8B1 promoter region in TCGA colorectal tumor and normal samples from the MEXPRESS. (COAD: 461 primary tumor samples a [file 2015648.f1.docx]

| **Approved**  **symbol** | **Chromosome** | **Transporter of menmbrane phospholipid** |
| --- | --- | --- |
| ATP8A1 | 4p13 | yes |
| ATP8A2 | 13q12.13 | yes |
| ATP8B1 | 18q21.31 | yes |
| ATP8B2 | 1q21.3 | yes |
| ATP8B3 | 19p13.3 | yes |
| ATP8B4 | 15q21.2 | yes |
| ATP9A | 20q13.2 | yes |
| ATP9B | 18q23 | yes |
| ATP10A | 15q12 | yes |
| ATP10B | 5q34 | yes |
| ATP10D | 4p12 | yes |
| ATP11A | 13q34 | yes |
| ATP11B | 3q26.33 | yes |
| ATP11C | Xq27.1 | yes |
| ABCA1 | 9q31.1 | yes |
| ABCA2 | 9q34.3 | yes |
| ABCA3 | 16p13.3 | yes |
| ABCB1 | 7q21.12 | yes |
| ABCB4 | 7q21.12 | yes |
| ABCB11 | 2q31.1 | yes |
| ABCC1 | 16p13.11 | yes |
| ABCG1 | 21q22.3 | yes |
| ABCG2 | 4q22.1 | yes |
| ABCG4 | 11q23.3 | yes |
| ABCG5/G8 | 2p21 | yes |
| PLSCR1 | 3q24 | yes |
| PLSCR2 | 3q24 | yes |
| PLSCR3 | 17p13.1 | yes |
| PLSCR4 | 3q24 | yes |
| Anot6 | 12q12 | yes |
| XKR8 | 1p35.3 | yes |

***Table S1: Gene lists of P4-ATPases, ABC transporters and Scramblases***

***Table S2 Patient demographics of DEGs analysis***

|  | **TCGA Colorectal cancer**  **N=382** | **TCGA Colorectal normal**  **N=51** |
| --- | --- | --- |
| Gender  male  female | 207 (54%)  175 (46%) | 27 (53%)  24 (47%) |
| Age (years)  median (IQR)  >65 vs ≤65 | 66 (56-75)  195 vs 187 | 70.5 (63-77)  30 vs 21 |
| Race  white  non-white  N/A | 275  73  34 | 21  16  14 |
| Stage  I  II  III  IV  N/A | 56  136  117  53  20 | -  -  -  -  - |

**Figure S1: A.** The differential expression of 31 genes for P4-ATPases, ABC transporters, and scramblases in matched TCGA colorectal tumor and normal samples (n=32) is shown in the –log (FDR) vs. log (FC) plot. The red dots represent 4 upregulated genes, the blue dots represent 5 downregulated genes, and the remaining black dots represent genes that are not differentially expressed. **B.** The different RNA *ATP8B1* expression between paired CRC tumor and normal samples. **C.** The different RNA *ATP8B1* expression between 44 CRC cell lines, 95 tumor and 60 normal tissues. **D.** The different protein spectral ATP8B1 count between 44 CRC cell lines, 95 tumor and 60 normal tissues.(the * means p<0.05 and **** means p<0.0001).

**Figure S2:** Subgroups analysis of OS based on gender, age, stage, KRAS mutation status, Braf mutation status, and TP53 mutation status in GSE39582 data.

**Figure S3:** Subgroups analysis of RFS based on gender, age, stage, KRAS mutation status, Braf mutation status, and TP53 mutation status in GSE39582 data.

**Figure S4: A.** A heatmap between *ATP8B1* expression and genetic alteration including copy number status of Chromosome 18q from the cBioportal (526 TCGA colorectal cancer samples). **B.** The relationship between *ATP8B1* copy number variations and gene expressions of 382 TCGA colorectal samples from the UCSC Xena browser (High-level Deletion: -2, Low-level Deletion: -1, Diploid: 0, Low-level Amplification: 1). **C.** The methylation level of the *ATP8B1* promoter region in TCGA colorectal tumor and normal samples from the MEXPRESS. (COAD: 461 primary tumor samples and 93 normal samples; READ: 172 primary tumor samples and 19 normal samples).

**Raw data:**
